# Automatic Grading of Retinal Blood Vessel in Deep Retinal Image Diagnosis

**DOI:** 10.1007/s10916-020-01635-1

**Published:** 2020-09-01

**Authors:** Debasis Maji, Arif Ahmed Sekh

**Affiliations:** 1 Haldia Institute of Technology, Haldia, India; 2 UiT The Arctic University of Norway, Tromsø, Norway

**Keywords:** Diabetic retinopathy (DR), Retinopathy of prematurity (ROP), Tortuosity-based grading

## Abstract

Automatic grading of retinal blood vessels from fundus image can be a useful tool for diagnosis, planning and treatment of eye. Automatic diagnosis of retinal images for early detection of glaucoma, stroke, and blindness is emerging in intelligent health care system. The method primarily depends on various abnormal signs, such as area of hard exudates, area of blood vessels, bifurcation points, texture, and entropies. The development of an automated screening system based on vessel width, tortuosity, and vessel branching are also used for grading. However, the automated method that directly can come to a decision by taking the fundus images got less attention. Detecting eye problems based on the tortuosity of the vessel from fundus images is a complicated task for opthalmologists. So automated grading algorithm using deep learning can be most valuable for grading retinal health. The aim of this work is to develop an automatic computer aided diagnosis system to solve the problem. This work approaches to achieve an automatic grading method that is opted using Convolutional Neural Network (CNN) model. In this work we have studied the state-of-the-art machine learning algorithms and proposed an attention network which can grade retinal images. The proposed method is validated on a public dataset EIARG1, which is only publicly available dataset for such task as per our knowledge.

## Introduction

**Fig. 1:**
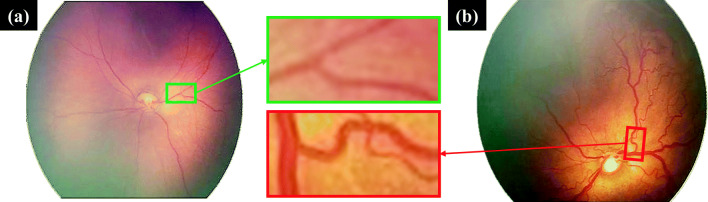
Example of **a** low tortusity fundus image **b** high tortusity image

The major cause of poor eye health is Diabetes. This is a dangerous malady, which not only affect the human eye but also the cause of several heart problems. In many cases the main cause of blindness is diabetic retinopathy (DR) [1, 2]. Diabetic retinopathy (die-uh-BET-ik ret-ih-NOP-uh-thee) is caused by long term diabetes that affects eyes. It’s caused by damaging the blood vessels of the light-sensitive tissue at the back of the eye (retina)[3]. In ophthalmology, retinal picture examination is a helpful instrument for the non-invasive determination of numerous important illnesses, for example, hypertension, diabetes or atherosclerosis. Basic side effects of those pathologies incorporate neovascularization, event of obsessive structures, or expanded tortuosity that can be watched examining the vascular tree of the eye fundus [4]. Early prognosis of DR can prevent many complicated health issues including blindness. The process begins with the analysis of rear of an eye; also known as the fundus. The images are captured using specialized fundus cameras consisting of an intricate microscope. The initial sign of DR includes the increment of the thickness of vessels and twisted vessels known as tortuosity [5]. For example, Fig. 1 presents a low tortusity and high tortusity fundus images.

As the vessels are so gaunt by nature, the chance of internal bleeding may occur [6]. So the measurement of the curvature of the vessels to detect DR is common for various ophthalmologist. Lotmar et al. [7] proposed a method based on chord and arc length and it becomes popular [8, 9]. However due to the difficulties in the measurement of proper curvature, Bullit et al. [10] and Grisan et al. [11] came with a modification, having same convexity, the vessels are grouped together with is a weighted summation. Curvature-based approach introduced by Hart et al. [12] was the first approach in this area. To measure of tortuosity, the integration is opted over squared curvature derivative [13]. Few works are proposed [14, 15] using curvature-based advent. Template disk is another method to measure the curvature of the vessel [4]. This process is very efficient but the computational burden is also high. Direction variation of the vessel is another way to measure the tortuosity. The fort value angle of sample centres coordinates are taken for the measurement [16, 17]. Signal processing approaches are also introduced in [18, 19]. The authors used Fourier analysis and Hough transform for the curvature measurement. The main drawback of vessel centric curvature measurement is the demand of vessel extraction from the fundus images. Non Subsampled Contourlet Transform (NSCT) is also used to calculate the curvature [20] which is not involving the vessel extraction step. Using spline operator, Wallace et al. [21] also avoid vessel extraction. Modeling based method [22], sine wave simulation and 3D modeling approach [23] is used in various application [24]. Edge detection based analysis [25] such as 3-edge detectors used in several fundus image analysis. Edge detectors were used in this way that the screening of enormous populace of patients can be repetitive and tedious since there is a deficiency of pros, robotized approach is financially savvy as it can possibly expand the efficiency of the ophthalmologists in such circumstances and optimal fused pair of edge detectors was investigated [26]. All these methods relay on the accurate extraction and measurement of tortuosity. The methods utilize the whole image is limited due to the complexity and availability of suitable dataset. Automated computerized system for the investigation of retinal pictures will be a support for the ophthalmologists or different specialists [27].

### Motivation and contributions

Automatic grading of fundus images for identification eye disease due to diabetic is emerging. State-of-the-art such grading methods use the tortusity (curve) and sometimes it need human interaction for selecting region-of-interest (ROI). The eye condition (grade) is predictable by analyzing fundus images, higher the grade higher the risk of blindness. This blindness which can caused by diabetic is a truly preventable via early detection of DR and timely treatment. For example, ROP is an eye disease that affects prematurely born infants. Normal retinal blood vessels are straight or gently curved. In case of DR and ROP, the blood vessels become tortuous, i.e., they take on a serpentine path. Curvature is a significant attribute in shape analysis in general and tortuosity evaluation in particular. Several works proposed recently, which exploit curvature information to measure tortuosity and grade accordingly. The automation of the system can be useful in computer aided detection (CAD). In this research, a study of the state-of-the-art classification methods for grading fundus images are presented. The main contributions are:

- **(i)** An automatic grading system of fundus images is proposed. The method uses a neural network that does not demands manual tortuosity measurement.
- **(ii)** An attention based neural network combined with a transfer learning approach is proposed for grading the health condition.

Rest of the paper is organized as follows. “Related work” presents related work on various fundus image classification and diagnosis methods. Proposed fundus image grading system is presented in “Proposed method”. Experimental results are discussed in “Experimental results”. “Discussion of results” presents the analysis of the results including time complexity and limitations. Finally, we conclude in “Conclusion”.

## Related work

Morphological change in the retinal vain structure often signifies the presence of some retinopathic disease [28]. Tortuosity in vessel structure is one of the important causes indicating proper treatment. In case of prematurity, the tortuosity of the vessel increases making the disease more severe. In case of Retinopathy of Prematurity (ROP), the development of the normal blood vessel in retina is suppressed by the growth of abnormal vessels. Diabetic Retinopathy (DR) have proved to have significant complication in premature infants having weight 1500 gram or less or born within a gestational age of 30 weeks [8]. DR if not diagnosed properly may lead to long-term vision loss or even blindness. The purpose of diagnosing ROP is ablation of the abnormal factors that favours the growth of new blood vessels in immature retina using cryotherapy [8] or laser treatment [8]. There are clinically established methods fro ROP classification [8]. The classification divides an eye in several zones [8] and the severity of the disease is analyzed based on three parameters: **(i)** the position of the zone where new vessels are located, **(ii)** how much area of the retina is involved (which is determined by dividing the retinal area into clock hours) and **(iii)** how much fibrosis is associated with the blood vessels. There also exist another parameter that is the presence of ’plus disease’, which is associated with ROP and is characterized by an increase in width and tortuosity of the retinal vessels. For evaluating tortuosity, a five level grading technique is proposed in [29] based on qualitative and subjective measure [10]. The severity of ROP is determined by counting the number of times a vessel has twisted and the amplitude of each twist [12]. It has been noted that with the increase in harshness also imply the increase of tortuosity [8]. Based on the curvature of retinal vessel, estimation of tortuosity has been carried out in [30]. An overview of the methods used in retinal vessel tortuosity calculation is reported in [24]. Chain coding technique has been used in [31] for tortuosity analysis. To quantify ROP, changes in width and tortuosity of blood vessel is analyzed in [8]. Chakravarty et al. [32] used quadratic polynomial decomposition method for tortuosity measurement. Machine learning technique has been adapted for measuring tortuosity exploring thickness and curvature based improved chain code in [33–35]. Latib et al. [36] proposed morphological approaches, where vessel partition is used for measuring the tortuosity. Post-processing is used in many cases to improve the accuracy. Haralick texture features based post-processing are popular combined with neural network [37]. Recent progress in deep neural network opened up new possibilities in various image processing tasks including fundus image grading. Sahlsten et al. [38] proposed a deep neural network for macular edema grading, A Graph Neural Network (GNN)-based method to improve accuracy for severity classification is proposed in [39], the method use interest regions for classifying severity. A quality-based grading system using interpretable deep learning is proposed in [40]. Bhattacharya et al. [41] proposed a health grading method based on the presence of red lesions. Fundus image quality assessment is proposed in [42], the method uses convolutions neural network (CNN) for the task. However, none of the method uses the dataset that use tortuosity for such grading. Mobile-based diagnostic systems is also used to examine large number of individuals with diabetes, it is useful where availability of ophthalmologists are limited [43].

ROP is a significant health problem which can be identified through screening test which is an economic and specific treatment for effectively treating of the disease. At present ophthalmologists skilled in examining infants’ eyes carry out the screening process. Automated system that can assist ophthalmologists in the screening process, or that can allow less trained individuals to execute the screening would be of clinical benefit. A possibility of providing some automated assistance in this screening process lies in accurate computer measurement of vessel width and tortuosity near the optic disc, which is situated near the posterior pole of the retina. This posterior pole can be easily visualize using a fundus camera and hence analyzing this region will be efficient during the screening test. This possibilities attract researchers to design suitable automated methods to assist ophthalmologists. State-of-the-art automated methods focused on segmentation, optic disc detection, and tortuosity detection. Hence, most of the publicly available datasets consists of manually labled vessel and optic disc. Table 1 summarized some public datasets and methods used in various fundus image analysis. Table 2 summarized different fundus image analysis methods and their limitations. Table 3 presents some key methods applied in various fundus image analysis method and the accuracy over different dataset.

**Table 1:** Popular fundus image datasets and applications

Sample Image	Dataset	Description
	HRF [44]	Task: Vessel segmentation
		Modality: Color Fundus
		Total Images: 15
		GT: Vessel, OD
	DRIVE[3, 45]	Task:vessel segmentation
		Modality:Color Fundus
		Total Images :40
		GT: Vessel
	STARE [44]	Task:vessel segmentation
		Modality:Color Fundus
		Total Images : 400 (overall), 40 (vessels)
		GT: Vessel, OD, EXUDATES,
		MICROANEURYSMS
	CHASE-DB1 [44]	Task:vessel segmentation
		Modality:Color Fundus
		Total Images :image L[14],image R[14]
		GT: Vessel
	DIARETDB1 [46]	Task: EXUDATES, MICROANEURYSMS,
		HEMORRHAGE
		Modality: Color Fundus
		Total Images :89
		GT: OD, EXUDATES,
		MICROANEURYSMS
	MESSIDOR [46]	Task: DR grading
		Modality: Color Fundus
		Total Images: 1200
		GT: EXUDATES,
		MICROANEURYSMS, OD
	RIM-ONE [37]	Task: detect the optic cup and optic disc
		Modality:Color Fundus
		Total Images :169
		GT: optic disc, OD
	INSPIRE-AVR [47]	Task: Classify and grade different complications of HR
		Modality: Color Fundus
		Total Images: 40
		GT: Vessel, tortusity grading
	EIARAG1 [4]	Task: Classify and grade different vessel tortusity
		Modality: Color Fundus
		Total Images :120
		GT: Vessel tortusity grading

**Table 2:** Different applications, datasets, and the limitations of fundus image analysis methods reported in the literature

Reference	Application	Dataset	Method	Limitation
[4, 5]	Vessel tortuosity Measurement	120-Full image from ROP	Curvature-Based Algorithm	Small database used for validation
[11, 18, 19]	Vessel tortuosity Measurement	60 retinal images	Monte carlo simulation	Required higher computational
		30 Arteries30 Veins	Rigid Transformations	cost in the process
[3, 45]	Segmentation of the retinal blood vessel			
	Glaucoma classification	DRIVE dataset	SVM classifier	Demands manual segmented label for the training
[1, 2, 12]	Assessment of vessel tortuosity	20 retinal image	Tukey’s method	Required manual preprocessing before using it
[6, 7, 48]	Glaucoma classification	Non-public dataset	K-nearestneighbours and SVM	Demands large volume training data
[23, 26]	Retinal vessels’ diameter measurement	Non public	Dempster-Shafer Fusion	Limited due to learning limitaions

**Table 3:** Different applications, datasets, and performance of fundus image analysis methods reported in the literature

Dataset	Applications	Methods	Accuracy
ROP [4]	Tortusity measures	Curvature-based	0.71 (SCC)
DRIVE, STARE,	BV segmentation	Deformable U-Net	Accuracy of 0.9566, 0.9641, 0.9610, 0.9651.
CHASE DB1,			AUC of 0.9802, 0.9832,
and HRF			0.9804,and 0.9831 on DRIVE,
			STARE, CHASE DB1 and HRF respectively
DRIVE, STARE,	Exudates	Mutual information:	Avg. accuracy of 0.9771
DIARETDB1,		Laplacian of Gaussian (LoG) filter,	and AUC of 0.9554 on
and MESSIDOR		matched filter (MF),	DRIVE, STARE,
		differential evolution (DE) algorithm	DIARETDB1, and MESSIDOR
RIM-ONE	Cup to Disc measurement for	Haralic features were applied	Accuracy of 0.8643
	Diagnosing Glaucomausing	and post-process using	
	Classification Paradigm	a neural network	
MESSIDOR	Macular edema in	Local binary patterns (LBPs),	Sensitivity of 0.925,
	Multispectral images (MSI)	generalized low-rank approximation	specificity of 0.983,
		of matrices (GLRAM), supervised	and accuracy of 0.981
		regularization term (SRT),	
		Gaussian kernel-based SVM	
DRIVE, INSPIRE-AVR	Graph-based classification	Classification of arteries and veins	0.874; 0.883 (ACC)

## Proposed method

**Fig. 2:**
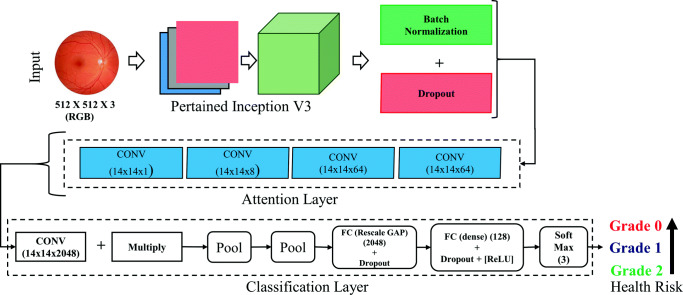
Overview of the proposed attention-based learning framework for grading fundus images. It consists of a pretrained model (Inception V3) and an attention network to classify the images in 3 grade, where higher grade represents higher risk of eye problems

In our proposed method, we design a optimize learning method for grading fundus images, where higher the grade higher chance of eye disease that may lead to blindness. The method can be used for early detection of such symptoms. It is noted that the vessel is one of the important area that is used to grade eye. We employ an attention network that add an attention over pixels near vessel for classification. The method uses Inception V3 for the feature used to estimate the attention. The overall method is depicted in Fig. 2.

The method sequentially uses prepossessing, augmentation, and the learning module. Firstly, to reduce the effect of lighting distinctions such as brightness and incident angle we employ filters. We have used a Gaussian filter to normalize the color balance as well as local illumination of fundus image. Next, the data is augmented using cropped randomly on both height and width, randomly mirrored images between 1 degree and 180 degree, and changing image ratio randomly between 0% and 15%. Finally, an attention network is considered for learning and grading.

**Fig. 3:**
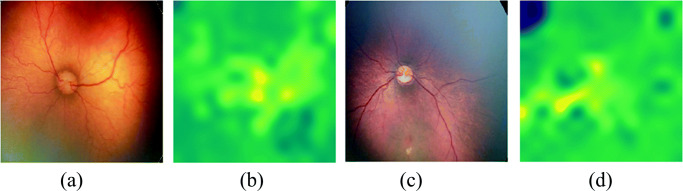
Example of attention on **a**–**b** low tortusity fundus image **c**–**d** high tortusity image

The major tasks of such automatic grading system are feature extraction and classification. Firstly, we have used transfer learning for the feature extraction. We have used Inception network pretrained on Kaggle competition dataset. The architecture uses traditional Convolution Network (CNN) for feature extraction and then fully connected and softmax for classification. Let, the fundus image (*I*), the role of feature extractor is to learn and extract suitable features. The use of transfer learning speed up the training process and also achieve higher accuracy. Next, the feature vector is passed through a simple attention layer that automatically hides areas that aren’t relevant for making the grading (here, the non-vessel area). Attention network is one of the popular methods in state-of-the-art machine learning. In various researches, attention is used for image classification, text classification, image captioning, etc. Here, we have used the attention for grading fundus images based on tortuosity. Let, *p* is a pixel of the *w* × *h* fundus image and *p* ∈ *I*_*w*×*h*_ and the feature vector (*z*) is extracted using pretrained model, *z* ∈ *I*_*k*_. Let, *a* = [0, 1]_*k*_ is attention vector. The attention view (*v*) is defined in Eq. 1, where *a* is extracted from an attention neural network with *θ* parameters (*f*_*θ*_(*x*))and ⊙ is element wise multiplication. For example, (Fig. 3) depicts such attention in grade 0 and 3 fundus images. Algorithm 1 presents the algorithm for the training procedure.

1 $$  v= a \odot z $$

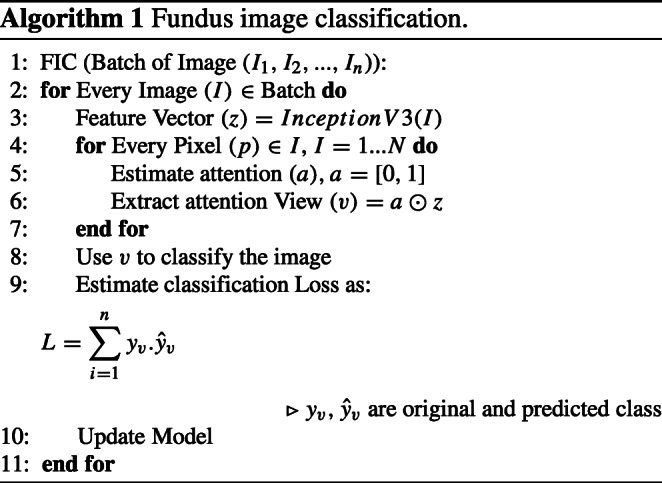

## Experimental results

Grading eye based on tortusity is challenging because of the complexity of the procedure. State-of-the-art machine learning algorithms needs specialize tuning to achieve satisfactory results. In this section, we present the performance of the state-of-the-art deep learning algorithms for grading. We come up with an attention network that performs satisfactorily. We have analyzed the attention framework extensively by varying several dependencies.

### Datasets

**Fig. 4:**
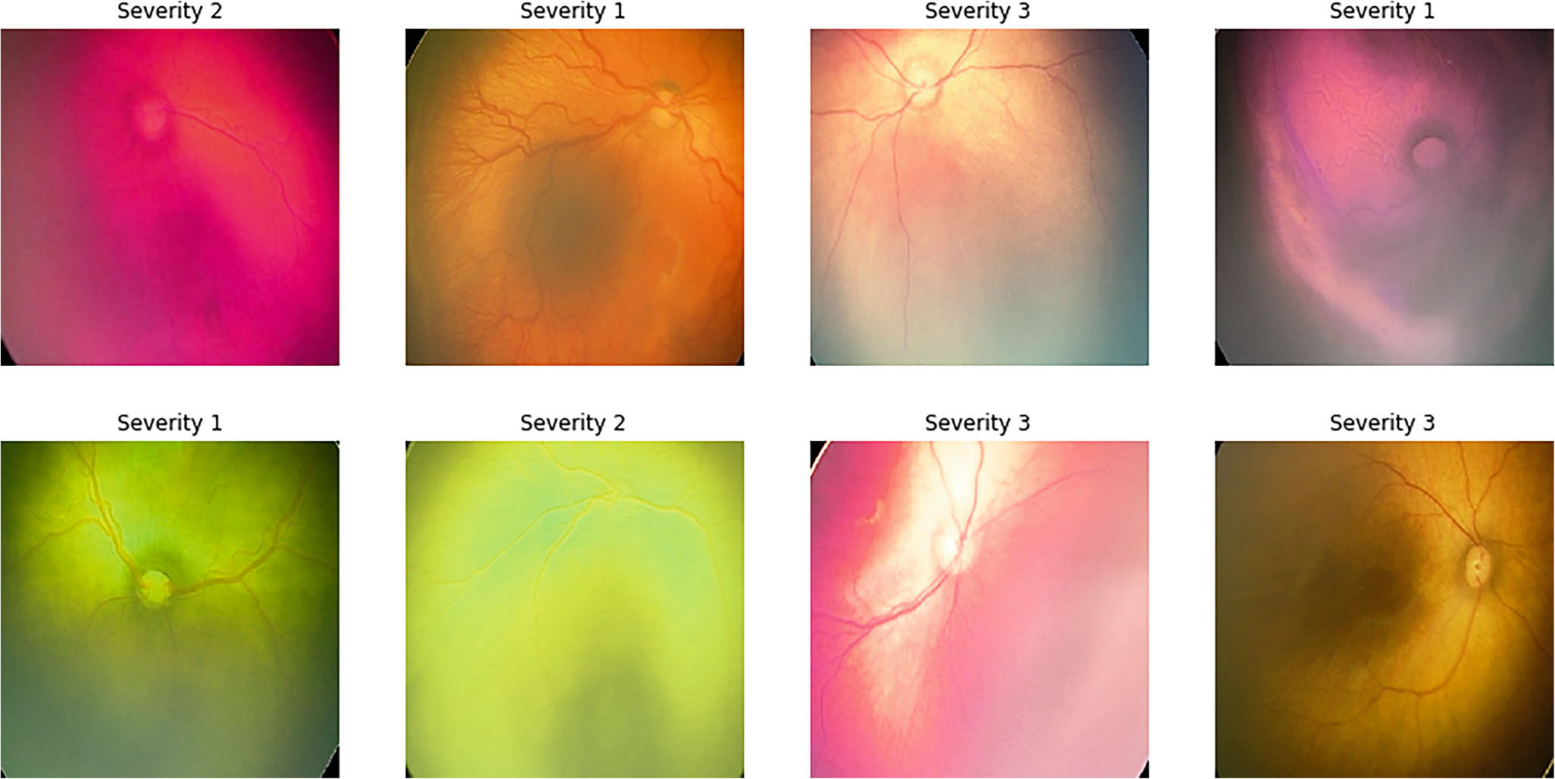
Visualizations of the augmented images used for training

Most of the state of the art datasets are design for optic disc detection, segmentation, and tortusity measurement (See Table 1). Although these are directly related to DR, the datasets are labeled into two classes namely healthy and non-healthy. Here, we proposed method for grading the health condition opposed with the binary classification used in the state-of-the-art datasets. This methods demands a dataset which is labeled into different grade based on the health conditions. We found that only one dataset (EIARG1) is suitable for the case. For our experiments we have used two datasets. The first one is taken from Kaggle database^1^ contains 88702 fundus images. All the images are used for finetune Inception V3.0 and create a pretrained model for feature extraction. The EIARG1 database [4] has been provided by the Eye Images Analysis Research Group for image based studies on retinal blood vessel tortuosity in diabetic retinopathy. The dataset is publicly available (www.eiarg.um.ac.ir) and primarily used for identifying tortuosity of ROP images. The dataset contains 120 retina images and is used to validate the proposed method. For training, we have augmented the images as described earlier. Figure 4 show example of augmented images used for training.

### Benchmarked results

The grading method is benchmarked using state-of-the-art classification methods. The methods are benchmarked on EIARG1 dataset by taking 70% data as training and rest 30% for testing. We have also used 10-fold cross-validation in each case. The F1 scare is presented in Table 4. F1 is calculated as: $2 \times \frac {precision \times recall}{precision + recall}$. We have used classical methods such as KNN, random forest, multiclass SVM. We have also explored deep neural networks such as conventional CNN including ResNet50 [49] and VGG16 [50], encoder-decoder framework. It is observed that the proposed attention model with the pretrained Inception outperform.

**Table 4:** Performance of the learning methods for grading fundus images

Method	F1 Score
KNN [51]	0.59
Random forest [52]	0.63
SVM [53]	0.61
CNN [54]	0.84
Encoder-Decoder [55]	0.79
ResNet-50 [49]	0.82
VGG16 [50]	0.71
Attention+ResNet-50 [49]	0.81
Attention+VGG16 [50]	0.67
Attention+Inception V3	0.92

Next, we have compared state-of-the-art non neural network based grading system with our proposed method. Table 5 summarized the results. It is observed that the proposed method also produces a state-of-the-art accuracy similar to the non neural network based grading systems that demand complex measurement metrics.

**Table 5:** Overall comparison with state-of-the-art non neural network based methods. The accuracy measure is retinopathy online challenge score (SCC)

Reference	Method	Database	#of images	Performance (SCC)
Grisan et al. [11]	Inflection-based	RET-TORT	60	0.949 (artery),
	measurement			0.853 (vein)
Lagali et al. [56]	Combination	Non-public	20	0.95
	of measures	dataset		
Oloumi et al. [57]	Angle-variation-based	Non-public	7	NA
	measurement	dataset		
Aslam et al. [58]	Curvature and vessel	DRIVE	20	NA
	width-based measurement			
Aghamohamadian et al. [59]	Curvature-based	RET-TORT	60	0.94
	measurement			
Aghamohamadian et al. [4]	Curvature-based	RET-TORT	120	0.71
	measurement			
Proposed Method	Attention network	RET-TORT	120	0.98

## Discussion of results

Here, we critically analyzed the benchmarked results and the results of proposed method. The dataset contains manually labeled grade by different experts. We have discussed the correlation among experts and with the predicted output. The time complexity and limitation of the method is also discussed here.

It is noted that the classical image classification method such as KNN, SVM, etc. failed because of the complexity of the images. The finer difference among different grade of fundus images need to amplify for achieving higher accuracy. Classical methods including popular deep neural network also failed because of limited availability of data and unsuitable parameters. It is noted that the attention based method performs comparatively better because of the weighted attention mechanism that enable to focus on the vessel with higher weight.

### Analysis of attention network results

**Fig. 5:**
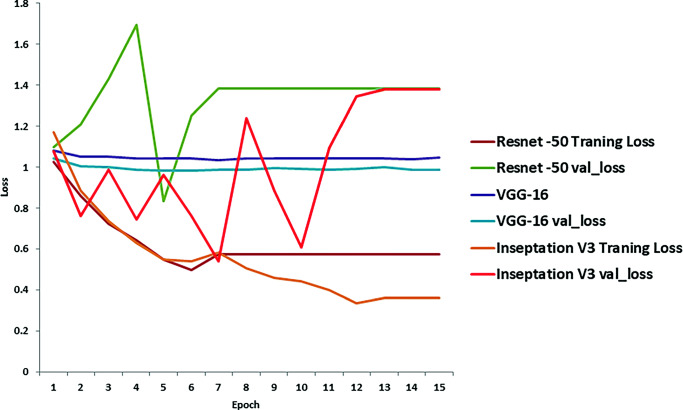
Training and validation loss during training using ResNet50 [49],VGG16 [50], and proposed attention and Inception [60]

In this section we present the results of the proposed method in details. The training loss and validation loss for the attention based models are presented in Fig. 5. It is observed that the proposed learning method also minimizes the loss during training and is much stable compared to ResNet50 and VGG16 backbone.

**Fig. 6:**
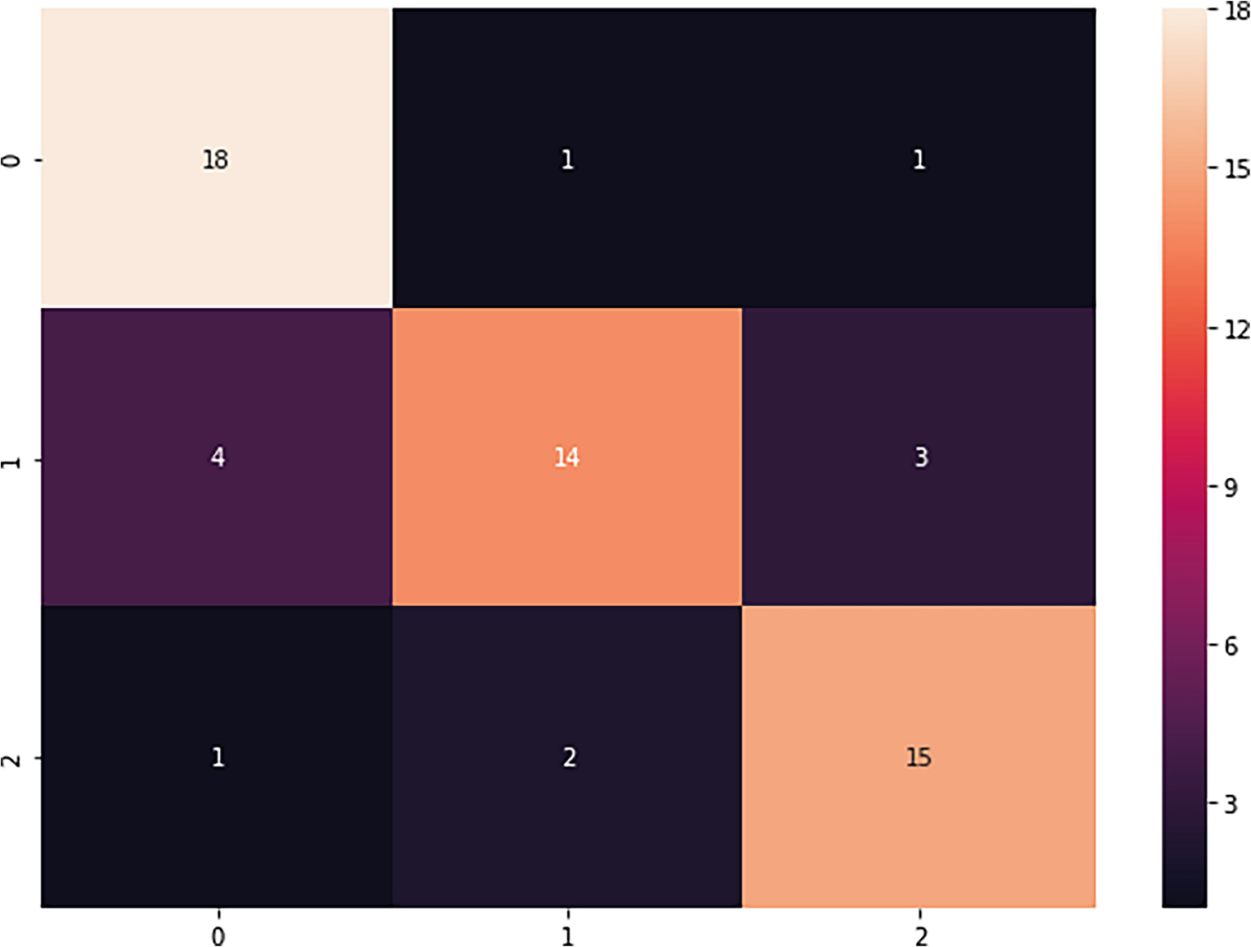
Confusion matrix of three grades in the test cases using the proposed method

Figure 6 depicts the confusion matrix of 3 classes (0,1,2) during validation. It is observed that the confusion among low tortuosity grades (0 are 1) is comparatively high than high tortuosity images (grade 3). Figure 8 shows example of successfully graded and wrongly graded fundus images using proposed method.

**Fig. 7:**
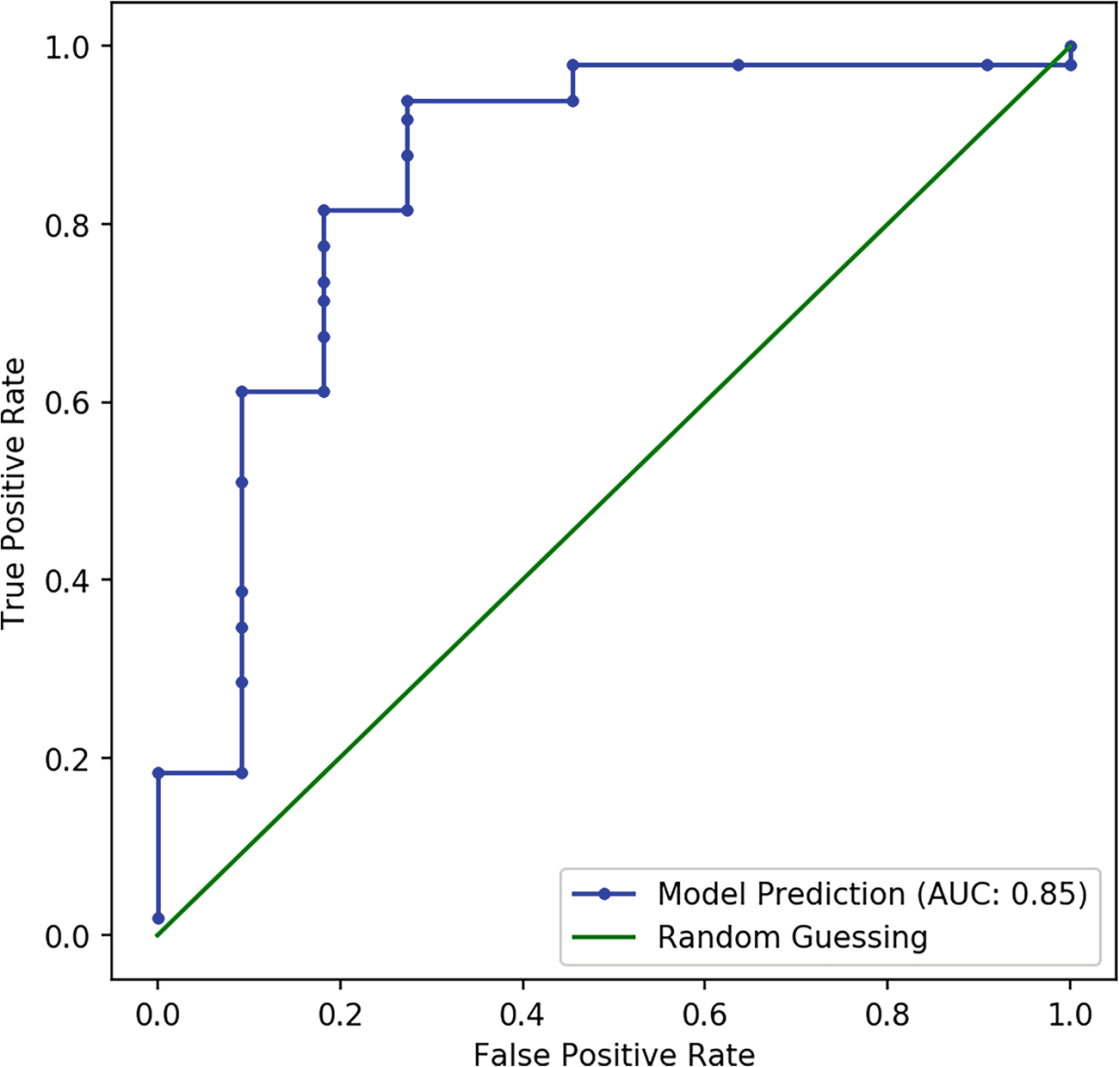
ROC curve for healthy V/s Sick

The proposed method outperform in case of finding sick/high risk fundus images. The receiver operating characteristic curve (ROC) is presented in Fig. 7. It is observed that the proposed method minimizes the false detection also.

### Correlation among experts

The grade of eye defines the possible risk of eye disease, it needs expert opinion. It is observed that same fundus image graded different by multiple doctors. Measuring tortuosity of the vessel is the state-of-the-art method for such grading. Automatic grading of fundus images reported in [4] is the baseline of the method.

**Fig. 8:**
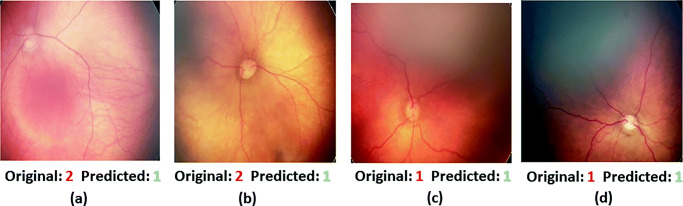
Some random example of failure cases **a**–**b** and successfully obtained grade **c**–**d** using proposed method

The method involves a 3-stage methods such as segmentation, key point identification, and tortuosity measurement. It uses 3 types of curvature measurements nonlinear estimation of curvature (*τ*_*nl*_), approximation of curvature (*τ*_*cp*_), and cross over angle (*τ*_*tr*_), which are complex and demands excessive training. We have estimated the correlation of each expert (doctors) with the automated method proposed in our method. Table 6 shows the results. It is observed that our proposed method ends with a higher correlation with experts. Figure 8 demonstrates some random successfully identified grades and failure cases.

**Table 6:** Correlation with the experts with the method [4] and the proposed method

Experts	*τ*_*nl*_	*τ*_*cp*_	*τ*_*tr*_	VGG16	Proposed
Exp. 1	0.61	0.56	0.41	0.52	0.70
Exp. 2	0.48	0.41	0.28	0.62	0.85
Exp. 3	0.7	0.61	0.48	0.72	0.98
Overall average vote	0.71	0.61	0.47	0.72	0.98

### Time complexity

**Fig. 9:**
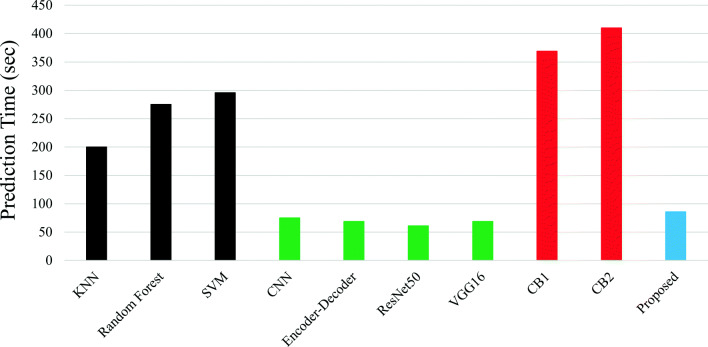
Execution time for 100 images. CB1 and CB2 are the curvature based methods reported in [59] and in [4]

The proposed method is a two steep process. First, the backbone of the method (Inception V3) is trained using Kaggle dataset and fine tune whole model using EIARG1. Next, the trained model is used to predict the grade. The experiments are conducted in Intel i7 processor (3.6 GHz), 16 GB of RAM, and NVIDIA Quadro P2000 GPU. The training takes ∼3 hours. Next, we have taken 10 random examples from the EIARG1 dataset for prediction and repeat the experiment 10 times (i.e. 100 predictions). Total prediction time taken by different methods are depicted in Fig. 9. It is observed that the proposed method takes similar time with the state-of-the-art deep models such as CNN and Encoder-Decoder based models. The method is computationally faster than the classical methods such as KNN and SVM and far better than the curvature based models.

### Limitations

The main drawback of the system is that it is validated on small dataset. Due the complex nature of grading fundus images. We found only one suitable dataset with a limited number of labelled images. It leads a small number of images to be trained and validated. Another limitation of the method is that the method blindly takes an image and predicts the grade. The method is not explainable like state-of-the-art vessel curvature based methods. Thirdly, the method is not really light weight model. It uses Inception V3 as backbone and Inception itself is a complex model. The proposed method demands special hardware such as high speed computer or GPU.

## Conclusion

Grading fundus images based on the tortuosity (vessel curve) is common in diabetic ratinopathy. It is complex and need special doctors. Automatic grading of such fundus images can be useful tool in various automatic diagnosis system. The method is in very early stage and only few datasets are available and number of samples in each grade is also very limited. State-of-the-art methods use segmentation of vessel to find the curvature of and grade the images which is difficult and performance is also low. In this paper, we have proposed deep-learning guided automatic grading of such images. We have used transfer learning for feature extraction where Inception V3 has been trained using 88702 fundus images and used for feature extraction. We propose an attention network for classify in 3 grades (high, medium, and low). The experiment shows that the proposed method outperforms compared to the state-of-the-art. The method also highly correlate with the experts. Automatic grading of eye based on the tortuosity demands intelligent methods. Combining other information such as age, sex, medical history can improve the detection.

## Footnotes

1 https://www.kaggle.com/c/diabetic-retinopathy-detection/data
